# Parallel Computation in Medical Imaging Applications

**DOI:** 10.1155/2011/840181

**Published:** 2012-02-23

**Authors:** Yasser M. Kadah, Khaled Z. Abd-Elmoniem, Aly A. Farag

**Affiliations:** ^1^Department of Biomedical Engineering, Cairo University, Giza 12613, Egypt; ^2^Biomedical and Metabolic Imaging Branch, NIDDK, National Institutes of Health, Bethesda, MD 20892-2560, USA; ^3^Department of Electrical and Computer Engineering, University of Louisville, Louisville, KY 40292, USA

There is currently a rapidly growing interest in parallel computation application in various medical imaging and image processing fields. This trend is expected to continue growing as more sophisticated and challenging medical imaging, image processing, and high-order data visualization problems are being addressed. The ongoing cost drop in computational tools and their wide accessibility play a center role as well. Given its short history, this area is still not a well-defined scientific discipline. The selected topics and papers for this special issue shed more light on various aspects of this expanding field and its potential in accelerating medical imaging applications. 

This special issue contains eleven papers covering various imaging modalities including MRI, CT, X-ray, US, and optical tomography. The papers demonstrated the potential of parallel computation in medical imaging and visualization in a wide range of applications including image reconstruction, image denoising, motion estimation, deformable registration, diffeomorphic mapping, and modeling.

In the paper entitled “*CUDA-accelerated geodesic ray-tracing for fiber tracking,*” E. van Aart et al. present an accelerated algorithm for brain fiber tracking. Noninvasive diffusion weighted imaging followed by reconstructing the brain fiber structure provides a unique way to inspect the complex structures inside the brain in a microscopic level. However, these processes are computationally expensive. The proposed algorithm utilizes the parallel structure of a graphics processing unit in combination with the CUDA platform to substantially accelerate the execution time of the fiber tracking by a factor up to 40 times compared to a multithreaded CPU implementation.

In the paper entitled “*Efficient probabilistic and geometric anatomical mapping using particle mesh approximation on GPUs,*” L. Ha et al. developed a new three-dimensional deformable registration algorithm for mapping brain datasets. The problem typically involves significant amount of computation time and thus became infeasible for practical purposes. The proposed registration method generates a mapping between anatomies represented as a multicompartment model. The implementation of the algorithm using particle mesh approximation on graphical processing units (GPUs) achieves the speed up of three orders of magnitudes compared to a CPU reference implementation, making it possible to use the technique in time-critical applications.

In the paper entitled “*Heterogeneous computing for vertebra detection and segmentation in X-ray images,*” F. Lecron et al. address the low computational efficiency of the conventional active shape model (ACM) algorithm and exploit the potential acceleration achieved when ACM is implemented on a parallel computation architecture. The paper demonstrates a global speedup ranging from 3 to 22, in comparison with the CPU implementation.

In the paper entitled “*Mapping iterative medical imaging algorithm on cell accelerator,*” M. Xu and P. Thulasiraman investigate the potential of parallel computation in accelerating the image algebraic reconstruction techniques which in one application may benefit image reconstruction on CT machines. The authors efficiently map the optimized algorithm on the cell broadband engine (BE) for improved performance over CPU version. The implementation on a cell BE is shown to be five times faster when compared to the performance on Sun Fire x4600, a shared memory machine.

In the paper entitled “*GPU-accelerated finite element method for modelling light transport in diffuse optical tomography,*” M. Schweiger introduces a GPU-accelerated finite element solver for the computation of light transport in scattering media. Solutions are presented for both time-domain and frequency-domain problems. A comparison with a CPU-based implementation shows significant performance gains of the graphics-accelerated solution, with improvements of approximately a factor of 10 and 20 for double- and single-precision computations, respectively.

In the area of MRI reconstruction, the paper entitled “*High-performance 3D compressive sensing MRI reconstruction using many-core architectures,*” by D. Kim et al., investigates how different throughput-oriented architectures can benefit compressed sensing (CS) MRI reconstruction algorithm and what levels of acceleration are feasible on different modern platforms. The authors demonstrate that a CUDA-based code running on a GPU can reconstruct a 256 × 160 × 80 volume from an 8-channel acquisition in as fast as 12 seconds, which is a significant improvement over the state of the art. This achievement may potentially bring CS methods even closer to clinical viability.

In the paper entitled “*True 4D image denoising on the GPU,*” A. Eklund et al. show the implementation of a four-dimensional denoising algorithm on a GPU. The algorithm was applied to a 4D CT heart dataset of the resolution 512 × 512 × 445 × 20. The result is that the GPU can complete the denoising in as fast as 8 minutes. On the contrary, the CPU implementation requires about 50 minutes. The short processing time increases the clinical value of true 4D image denoising significantly.

In the field of simulation and phantom modeling, the paper entitled “*Patient specific dosimetry phantoms using multichannel LDDMM of the whole body,*” by D. J. Tward et al., describes an accelerated automated procedure for creating detailed patient specific pediatric dosimetry phantoms from a small set of segmented organs in a child's CT scan. The algorithm involves full body mappings from adult template to pediatric images using multichannel large deformation diffeomorphic metric mapping with a parallel implementation. The performance of the algorithm was validated on a set of 24 male and 18 female pediatric patients. Running times for the various patients examined ranged from over 30 hours on a single processor to under 1 hour on 24 processors in parallel.

In the paper entitled “*Numerical solution of diffusion models in biomedical imaging on multicore processors,*” L. D'Amore et al. address the solution of nonlinear partial differential equations (PDEs) of diffusion/advection type, underlying most problems in image analysis. As a case study, the paper addresses the segmentation of medical structures and performs a comparative study of numerical algorithms arising from using the semi-implicit and the fully implicit discretization schemes. Comparison criteria take into account both the accuracy and the efficiency of the algorithms including convergence, execution time, and parallel efficiency. This analysis is carried out in a multicore-based parallel computing environment.

In the paper entitled “*On the usage of GPUs for efficient motion estimation in medical image sequences,*” J. Thiyagalingam et al. investigate the mapping of an enhanced motion estimation algorithm to novel GPU architectures. Using a database of three-dimensional ultrasound image sequences, the authors show that the mapping leads to substantial performance gains, up to a factor of 60 and can provide near-real-time performance. The paper also shows how architectural peculiarities of these devices can be best exploited in the benefit of algorithms, most specifically for addressing the challenges related to their access patterns and different memory configurations. The paper further evaluates the performance of the algorithm on three different GPU architectures and performs a comprehensive analysis of the results.

In the paper entitled “*Fast random permutation tests enable objective evaluation of methods for single subject fMRI analysis*” by A. Eklund et al., it is shown that how the computational power of cost-efficient GPUs can be used to speed up random permutation tests. These tests are commonly involved in fMRI data analysis for identifying areas in the brain that are active. However, the computational burden with processing times ranging from hours to days has made them impractical for routine use in single-subject fMRI analysis. A test on GPU with 10000 permutations takes less than a minute, making statistical analysis of advanced detection methods in fMRI practically feasible. To exemplify the permutation-based approach, brain activity maps generated by the general linear model (GLM) and canonical correlation analysis (CCA) are compared at the same significance level.

